# Simultaneous quantification of multiple endogenous biothiols in single living cells by plasmonic Raman probes†
†Electronic supplementary information (ESI) available: The synthetic route, ^1^H NMR spectra, ^13^C NMR spectra, and LC-MS spectra of the Raman reporter, the TEM micrograph and UV-vis extinction spectra of AuNSs, the Raman spectra of Cys, Hcy and GSH modified AuNSs/PEG, the Raman spectra of PRPs reacted with different concentrations of Cys, Hcy or/and GSH over time, the scatter plot of the PC1 *vs.* PC2 scores of the Cys product, Hcy product, GSH product and mixed product, the calibration curve for three constituents and two constituents, the changes of ΔC≡N in the presence of diverse amino acids, the real-time Raman spectra of PRPs reacted with Cys, Hcy and GSH in cell lysis, the Raman spectra of three product samples, and the localized electronic field of AuNSs against distance. See DOI: 10.1039/c7sc03218h


**DOI:** 10.1039/c7sc03218h

**Published:** 2017-08-29

**Authors:** Shan-Shan Li, Qi-Yuan Guan, Mengmeng Zheng, Yu-Qi Wang, Deju Ye, Bin Kang, Jing-Juan Xu, Hong-Yuan Chen

**Affiliations:** a State Key Laboratory of Analytical Chemistry for Life Science , Collaborative Innovation Center of Chemistry for Life Sciences , School of Chemistry and Chemical Engineering , Nanjing University , 210023 , China . Email: binkang@nju.edu.cn ; Email: xujj@nju.edu.cn ; Email: hychen@nju.edu.cn

## Abstract

Intracellular biothiols mediate many important physiological and pathological processes. Due to their low content and competing thiol-reactivity, it is still an unmet challenge to quantify them within a complicated intracellular environment. Herein, we demonstrated a strategy to discriminate three biothiols, *i.e.* cysteine (Cys), homo-cysteine (Hcy) and glutathione (GSH), and quantify their concentrations within single living cells, using one platform of Raman probe. By monitoring the reaction kinetics of biothiols with Raman probes and discriminating their products with a quantitative principal component analysis (qPCA) method, these three biothiols could be simultaneously quantified in both cell lysis and single living cells. The concentrations of Cys, Hcy and GSH in single Hela cells were 158 ± 19 μM, 546 ± 67 μM and 5.07 ± 0.62 mM, respectively, which gives the precise concentrations of these three biothiols at a single cell level for the first time. This method provides a general strategy for discriminating each component from a mixed system and has potential for quantifying any biomolecules within an *in vitro* or *in vivo* biological environment.

## Abstract 2

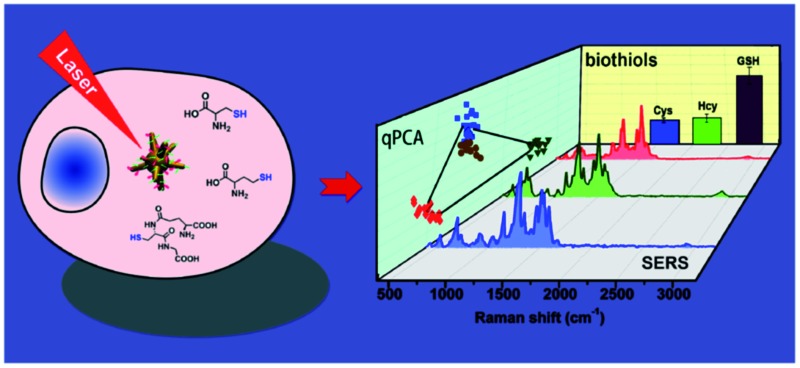
Three endogenous biothiols in single cells were simultaneously quantified by plasmonic Raman probes and quantitative principal component analysis (qPCA).

## Introduction

Biothiols, including cysteine (Cys), homo-cysteine (Hcy) and glutathione (GSH), are involved in many physiological and pathological events.^1^–^3^ The abnormal level of these biothiols within cells has been found to correlate with many health problems, such as liver damage, Alzheimer’s disease, cancer, coronary heart disease, AIDS and other syndromes.^4^–^8^ Measuring the expression level of endogenous biothiols in single living cells will definitely provide extremely valuable information about cell function, physiological regulation and disease development. However, simultaneously quantifying these biothiols within single cells still remains a challenge, due to their low content (*i.e.* μM–mM in <pL volume), competing thiol-reactivity, and also the complexity of the intracellular environment.

Over the past few decades, a number of techniques have been exploited to detect biothiols, including high performance liquid chromatography (HPLC),^9^,^10^ mass spectrometry (MS),^11^,^12^ capillary electrophoresis,^13^,^14^ electrochemistry assay,^15^,^16^ UV-vis absorption spectrometry,^17^,^18^ surface-enhanced Raman scattering (SERS),^19^–^21^ and fluorescence methods.^8^,^22^–^25^ Among these methods, fluorescence based imaging methods are the most available techniques for the detection of biothiols in cell samples. Several unique fluorescence probes have been designed to react with one or more types of biothiol, and thereby different biothiols could be measured through the alternation of fluorescence in different channels.^22^–^28^ Though fluorescence based imaging has many advantages, its potentials are restricted by the photobleaching of fluorophores, as well as spectral overlap between wide fluorescence bands (*i.e.* 100–200 nm or more). Comparatively speaking, Raman spectroscopy with a very narrow bandwidth (*i.e.* 1–2 nm) and a wealth of fingerprint information can provide much more molecular structural information.^29^,^30^ Thus far, only a few Raman-based biothiol detection methods have been reported,^19^–^21^ and two main challenges still remain unaddressed: discriminating different constituents within a multicomponent cellular system and quantifying the concentration of each constituent. Herein, we solved these two challenges by designing a Raman probe that could react with Cys, Hcy and GSH, and chemical reaction kinetics and quantitative principal component analysis (qPCA) allowed us to discriminate and quantify these three biothiols at the single cell level simultaneously *via* the one probe platform.

## Results and discussion

### Design of plasmonic Raman probes (PRPs)

**Fig. 1:**
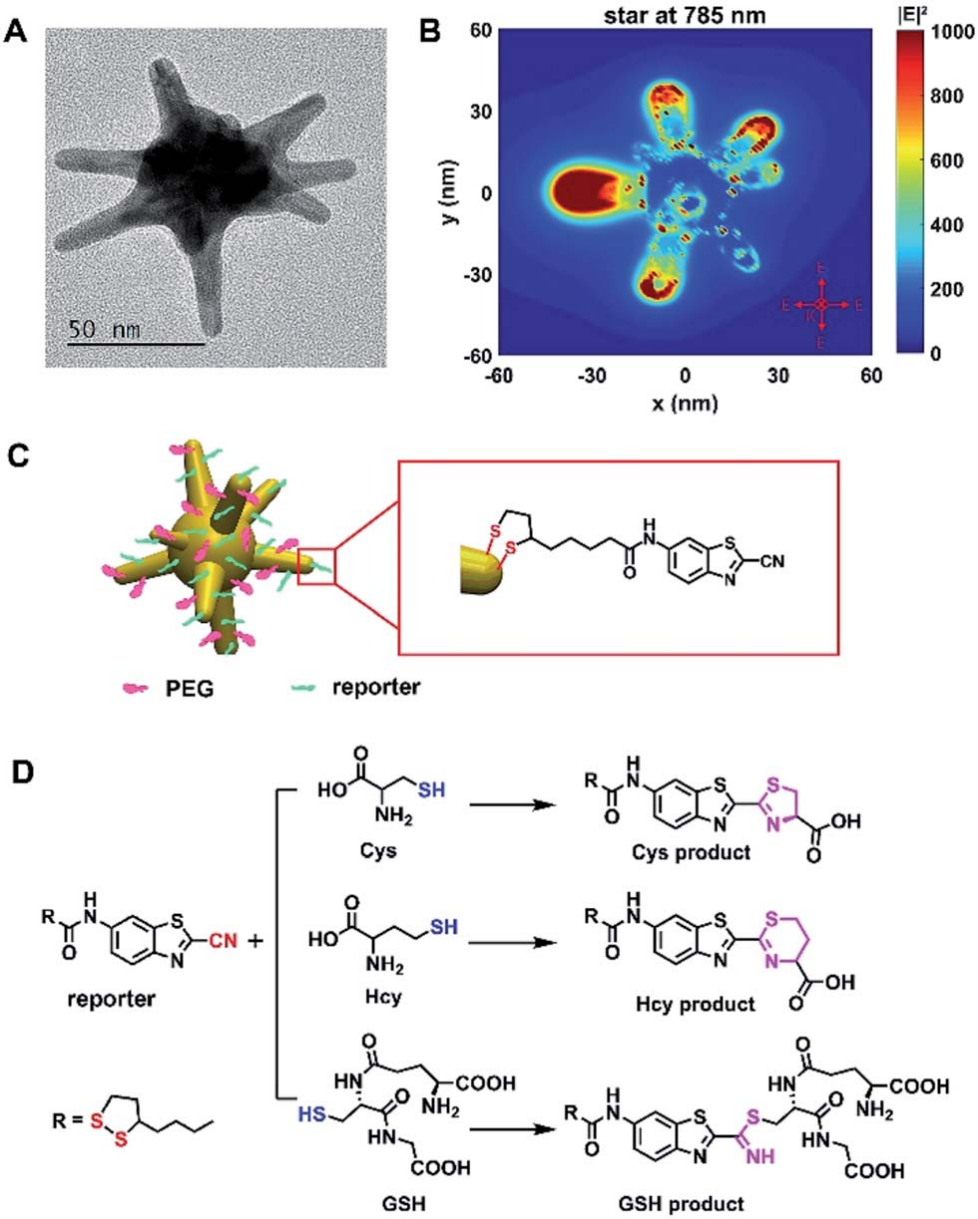
The design of plasmonic Raman probes (PRPs). (A) The transmission electron microscopy (TEM) micrograph of a gold nanostar (AuNS). (B) The Finite-Difference Time-Domain (FDTD) simulated distribution of the localized electronic field of the AuNS at 785 nm. (C) An illustration of the AuNS conjugated to PEG and the Raman reporter denoted as plasmonic Raman probes (PRPs). (D) The reaction mechanism of PRPs with Cys, Hcy and GSH.

The Raman probe was composed of polyethyleneglycol (PEG)-modified 60 nm gold nanostars (AuNSs) and Raman reporter molecules, denoted as plasmonic Raman probes (PRPs) (Fig. 1 and S1 and Table S1†). Scheme S1† depicts the synthesis route of the Raman reporter (*N*-(2-cyanobenzo[*d*]thiazol-6-yl)-5-(1,2-dithiolan-3-yl)pentanamide, TA-CBT) obtained by 2-cyano-6-aminobenzothiazole (CBT-NH_2_) and thioctic acid (TA) *via* a one-pot procedure, and the chemical structure was fully characterized by ^1^H/^13^C NMR (nuclear magnetic resonance) spectroscopy and liquid chromatography-mass spectrometry (LC-MS) analysis (Fig. S2–S4†). –CBT, as a biothiol probe, has previously demonstrated that it could selectively react with 1,2 and 1,3-aminothiol substrates (*e.g.* Cys and Hcy) with high reactivity, but it reacts with free thiol groups (*e.g.* GSH) with much lower reactivity.^31^,^32^ Considering the concentration of GSH is orders of magnitude higher than that of Cys and Hcy within the cellular environment,^23^,^26^,^33^–^36^ the unique selective reactivity of –CBT with Cys, Hcy and GSH offers us the opportunity to detect all three of the biothiols simultaneously, even when their intracellular concentrations are orders of magnitude different. Herein, within the structure of the Raman reporter TA-CBT, the five-membered ring containing a disulfide bond could be conjugated with AuNSs tightly avoiding displacement by intracellular sulfydryl, and the cyano-group (C≡N) can react with the sulfydryl groups (S–H) of the biothiols to form products with alternative structures (Fig. S5–S7† and 1D). The Raman spectrum of the PRPs was similar to that of the reporter with C≡N at 2235 cm^–1^,^37^,^38^ but different from those of the PEG–AuNSs, PEG and three biothiol modified AuNSs (Fig. S8A and S9A†). The Raman spectra of the three products showed the disappearance of C≡N at 2235 cm^–1^ and S–H at ∼2543 cm^–1^,^19^,^37^–^39^ which are quite different from those of Cys, Hcy, GSH and PRPs, suggesting that the reaction did happen (Fig. S8, S9B and C†).

### Strategy for discriminating and quantifying Cys, Hcy and GSH

**Fig. 2:**
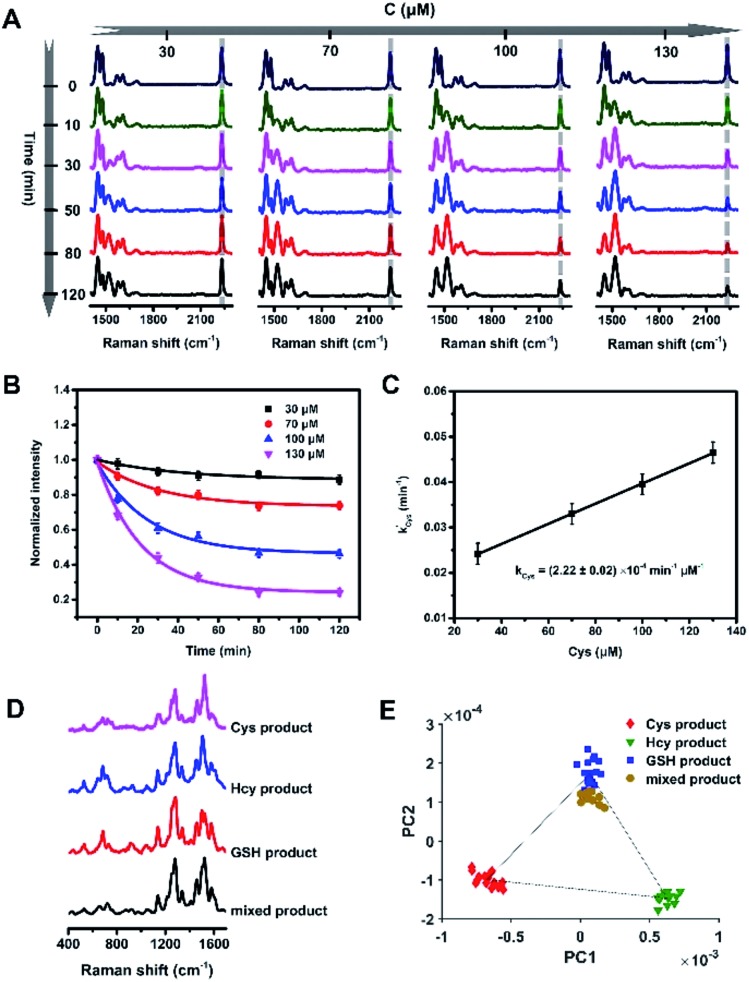
Strategy for discriminating and quantifying the biothiols. (A) The Raman spectra of PRPs reacted with different concentrations (30, 70, 100 and 130 μM) of Cys in pH 7.4 PBS at 37 °C for 120 min. (B) The trends of C≡N over time at different concentrations of Cys. (C) The pseudo first-order rate constant against the concentration of Cys. The original data for Hcy and GSH are included in the ESI.† (D) The Raman spectra of the three products and a mixed product in 400–1700 cm^–1^. (E) The scatter plot of the PC1 *vs.* PC2 scores of the corresponding four products.

As mentioned above, PRPs could react with the three biothiols and form three products with different molecular structures, which could be detected by Raman spectroscopy. This design offers possibilities for discriminating and quantifying these three biothiols simultaneously. To achieve this purpose, we developed a strategy combining reaction kinetics with the qPCA method (see the ESI† for details). Briefly, we consider the reactions of tiny PRPs with a relatively large excess of biothiols, its rate equation could be written as: *k*′_mix_ = [*k*_Cys_ + *k*_Hcy_(*y*/*x*) + *k*_GSH_(*z*/*x*)][Cys]_0_, where *k*′_mix_ is the pseudo-first-order reaction rate constant of a mixture with unknown [Cys]_0_, [Hcy]_0_ and [GSH]_0_, *k*_Cys_, *k*_Hys_ and *k*_GSH_ are the second-order rate constants for Cys, Hcy and GSH, respectively, and [Cys]_0_ : [Hcy]_0_ : [GSH]_0_ = *x* : *y* : *z*. From the above equation, [Cys]_0_, [Hcy]_0_ and [GSH]_0_ could be calculated if *k*′_mix_ and *x* : *y* : *z* were given, since *k*_Cys_, *k*_Hys_ and *k*_GSH_ do not depend on the concentration of the reactants. In this work, *k*′_mix_ could be experimentally determined by monitoring the reaction kinetics of PRPs with a mixture of Cys, Hcy and GSH, and *x* : *y* : *z* was obtained by running a qPCA analysis on the Raman spectra of their products (see the ESI† for details). When the PRPs reacted with Cys, Hcy or GSH, the C≡N in the PRPs disappeared gradually with the extension of the reaction time, so we could monitor the reaction kinetics by following the Raman band intensity of C≡N. Firstly, we examined the kinetic changes of the reaction with a relatively large excess of Cys, Hcy or GSH over PRPs in pH 7.4 PBS at 37 °C. With varying concentrations of Cys, Hcy or GSH, we collected the real-time Raman spectra of PRPs at different time intervals (Fig. 2A, S10A and S11A†). The pseudo-first-order reaction rate constant (*k*′) was calculated on the basis of the exponential fitting of C≡N over time (Fig. 2B, S10B and S11B†), which is proportional to the second-order reaction rate constant (*k*), and then we could calculate the second-order reaction rate constants for the reaction of PRPs with Cys, Hcy and GSH: (2.22 ± 0.02) × 10^–4^ min^–1^ μM^–1^, (1.74 ± 0.14) × 10^–4^ min^–1^ μM^–1^, and (3.53 ± 0.49) × 10^–6^ min^–1^ μM^–1^, respectively (Fig. 2C, S10C and S11C†). The reaction rates of PRPs with Cys and Hcy were about two orders of magnitude higher than the rate with GSH, which provided a possibility to discriminate low concentration Cys and Hcy (∼μM level) from high concentration GSH (∼mM level)^23^,^26^,^33^–^36^ according to their products. The Raman spectra of the end-products include abundant molecular structural information, but it is difficult to differentiate one from the other visually (Fig. 2D, S9B and C†). To extract the most principal differences, we analyzed the data by a qPCA method we proposed. Based on the 2D scatter plots (Fig. 2E, Tables S2 and S3 and Fig. S12 and S13†), we could calculate the weight of each of the products (*x* : *y* : *z*) in a mixed system (see the ESI† for calculation details).

If *k*′_mix_ and *x* : *y* : *z* were obtained, the initial concentrations of Cys, Hcy and GSH could be calculated *via* the workflow in Scheme S2.† We firstly verified the feasibility of this strategy *in vitro* in pH 7.4 PBS at 37 °C to simulate a cellular environment. According to the intracellular concentrations of Cys (30–200 μM), Hcy (0.1–1 mM) and GSH (1–10 mM) reported in previous literature,^23^,^26^,^33^–^36^ we carried out a series of simulation experiments. The pseudo-first-order reaction rate constants of the mixtures were obtained by fitting the changes of C≡N over time (Fig. S14–S16†), and the initial concentration percentages of the biothiols were calculated *via* qPCA (Tables S2 and S3†). Thereby, the calculated initial concentrations were obtained, with an average relative error of <15%, which were comparable with the given initial concentrations (Tables 1, S4 and S5†). The reaction selectivity of the current PRPs was also confirmed by a series of control experiments using biologically related amino acids and possible nucleophiles within a cellular environment (Fig. S17†).

**Table 1:** The simulation experimental calculated results *in vitro*

No.	Given concentration			Measured concentration		
	Cys (μM)	Hcy (μM)	GSH (mM)	Cys (μM)	Hcy (μM)	GSH (mM)
1	36	174	1.50	31	153	1.19
2	36	174	3.11	32	152	2.42
3	52	268	2.46	49	251	2.05
4	52	268	5.04	51	257	4.29
5	105	506	4.89	99	475	4.07
6	105	708	4.89	86	648	4.89
Average relative error (%)				9.42	8.29	15.19

### Discrimination and quantification of intracellular biothiols

**Fig. 3:**
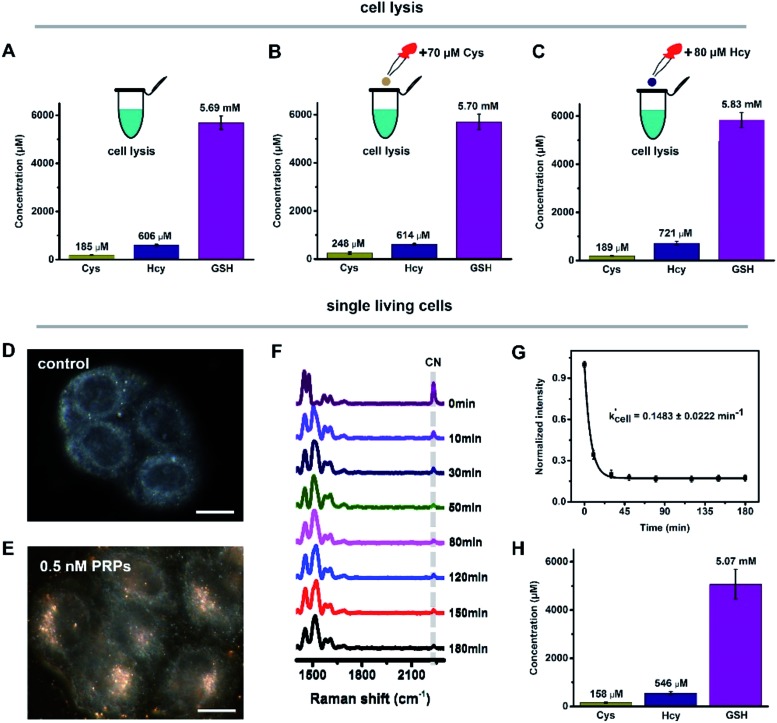
The discrimination and quantification of the intracellular biothiols. The concentrations of Cys, Hcy and GSH in cell lysis (A), and with the addition of 70 μM Cys (B) or 80 μM Hcy (C) in cell lysis. The dark field images of the Hela cells pre-incubated without (D) and with (E) 0.5 nM PRPs. The scale bar corresponds to 20 μm. (F) The Raman spectra of PRPs in single living cells over 180 min. (G) The trends of C≡N in cells against time. (H) The concentrations of Cys, Hcy and GSH in single living cells.

We then utilized this strategy to discriminate and quantify intracellular biothiols in cell lysis and single living cells. The real-time Raman spectra of PRPs reacted with Cys, Hcy and GSH in HeLa cell lysis were obtained (Fig. S18A†) and the pseudo-first-order reaction rate constant (*k*′_lysis_) was calculated by fitting the changes of C≡N over time (Fig. S18B†). Similarly, with the addition of 70 μM Cys or 80 μM Hcy into cell lysis to change the initial concentrations, *k*′_lysis_ was also acquired (Fig. S18C–F†). With the obtained *k*′_lysis_ and the weight of each component by qPCA, the initial concentrations of Cys, Hcy and GSH in HeLa cell lysis were 185 ± 9.1 μM, 606 ± 30 μM and 5.69 ± 0.28 mM (Fig. 3A–C), which are comparable to previous reports.^23^,^26^,^33^–^36^ Compared with cell lysis containing large numbers of cells, quantifying biothiols within a single cell was much more difficult. In our current system, the internalized PRPs were mainly localized in the cytoplasmic region of the HeLa cells (Fig. 3D and E), which enables the detection of the average level of biothiols within the cytoplasmic region, including cytosol and vesicular structures like endosomes and lysosomes. By monitoring the C≡N changes over time in the real-time Raman spectra of PRPs reacted with biothiols from single living cells (Fig. 3F), the reaction rate constant of single cells (*k*′_cell_) was calculated (Fig. 3G). Combining with the calculated concentration percentages, the initial concentrations of Cys, Hcy and GSH in the single HeLa cells were 158 ± 19 μM, 546 ± 67 μM and 5.07 ± 0.62 mM (Fig. 3H). These results were a little bit lower than those in cell lysis, which might be because the concentrations from cell lysis were the averaged content of total cells, but for single living cells, the detected concentrations were mainly from the cytoplasmic region, where the PRPs are located. Some subcellular organelles, such as mitochondrion,^27^ might contain a relatively high concentration of biothiols, which current PRPs cannot target. It is notable that the Raman spectra obtained from single cells and cell lysis were quite similar to those of the *in vitro* simulation experiments, indicating that the signal enhancement is mainly derived from the Raman reporters (Fig. S19†).

## Conclusions

We demonstrated a strategy to discriminate and quantify three endogenous biothiols within single cells by one platform of Raman probe. For the first time, the precise concentrations of Cys, Hcy and GSH were simultaneously obtained at the single cell level. This strategy combining reaction kinetics with the unique qPCA method provides potential for extracting and measuring biomolecules in complex biological environments, *i.e.* blood, tissue or even *in vivo* systems, which might offer insight into the real and practical applications including health care and clinical diagnosis. Limited by the sensitivity and spatial resolution of our Raman instrument, our current work only demonstrated the averaged value of biothiols from the whole area of the cytoplasmic region, instead of from different subcellular structures. With a high speed Raman mapping instrument, our current method could be further empowered and the biothiols within different subcellular organelles (*e.g.* early/late endosome, lysosome, mitochondrion, nucleus, and so on) are expected to be precisely revealed in the near future.

## Experimental section

### Chemicals

Isobutyl chloroformate, thioctic acid, 4-methylmorpholine (MMP), tetrahydrofuran (THF), 2-cyano-6-aminobenzothiazole, tetrachloroauric acid trihydrate (HAuCl_4_·3H_2_O), cysteine (Cys), homocysteine (Hcy) and glutathione (GSH) were purchased from Sigma-Aldrich (Shanghai, China). Silver nitrate (AgNO_3_), hydrochloric acid (HCl), ascorbic acid (AA), and trisodium citrate were purchased from Aladdin Chemistry Co. Ltd (Shanghai, China). Polyethyleneglycol (PEG, MW 5000) was obtained from Jenkem Technology Co. Ltd (Beijing, China). All chemicals were used without further purification, unless otherwise stated.

### Synthesis of gold nanostars (AuNSs)

AuNSs were synthesized by a seed-mediated growth method.^40^ For seed preparation, 15 mL of 1% trisodium citrate solution was added to 100 mL of boiling 1 mM HAuCl_4_ solution under vigorous stirring for 15 min. For AuNS synthesis, 0.5 mL of the above seed solution (∼13 nm, *A* = 2.81) and 50 μL of 1 M HCl were added to 50 mL of 0.25 mM HAuCl_4_ solution at room temperature under moderate stirring. Then, 1 mL of 0.5 mM AgNO_3_ and 0.25 mL of 0.1 M AA were added simultaneously and then stirred for 30 s.

### Synthesis of Raman reporter

Isobutyl chloroformate (204 mg, 1.5 mmol) was added to a mixture of thioctic acid (309 mg, 1.5 mmol) and MMP (4-methylmorpholine, 202 mg, 2.0 mmol) in THF at 0 °C. The mixture was stirred for 30 min, to which 2-cyano-6-aminobenzothiazole (175 mg, 1.0 mmol) was then added, and then stirred at room temperature overnight. After the reaction, the solvent was removed under vacuum, and the residue was purified by flash chromatography on silica gel to yield the desired product Raman reporter as a white yellow solid (268 mg, 74%). ^1^H NMR (400 MHz, DMSO-*d*_6_) *δ* 10.40 (s, 1H), 8.76 (d, *J* = 2.0 Hz, 1H), 8.18 (d, *J* = 8.0 Hz, 1H), 7.72 (dd, *J* = 2.0, 8.0 Hz, 1H), 3.64 (m, 1H), 3.16 (m, 2H), 2.42 (m, 3H), 1.88 (m, 1H), 1.65 (m, 4H), 1.44 (m, 2H); ^13^C NMR (100 MHz, DMSO-*d*_6_) *δ* 171.75, 147.39, 139.76, 136.71, 134.70, 124.71, 120.58, 113.58, 110.92, 56.05, 38.08, 36.28, 34.13, 28.28, 24.73. LC-MS: *m*/*z* calcd for C_16_H_18_N_3_OS_3_^+^ [(M + H)^+^]: 364.06; found 364.00. The details are shown in Scheme S1 and Fig. S2–S4.†

### Preparation of plasmonic Raman probes (PRPs)

The AuNSs (50 mL of 0.1 nM) were first incubated with 100 μL of 0.05 mM PEG in a shaker for 12 h and then purified by centrifugation at 8000 rpm for 10 min. Subsequently, these PEGylated AuNSs (4 mL of 0.5 nM) were treated with 200 μL of 0.05 mM reporter and purified using centrifugation at 8000 rpm for 10 min to yield plasmonic Raman probes (PRPs). These PRPs were re-dispersed in phosphate-buffered saline (PBS, 1×, pH 7.4) for subsequent use and kept at 4 °C for long-term storage.

### Preparation of samples for SERS measurement

The three products (Cys product, Hcy product or GSH product) were obtained by reaction with a large excess of Cys, Hcy or GSH over PRPs in pH 7.4 PBS at 37 °C. By varying the concentrations of Cys, Hcy or GSH reacted with PRPs, a series of samples were prepared in pH 7.4 PBS at 37 °C. Multiple mixed products were gained *via* the reaction with different concentrations of Cys, Hcy and/or GSH in pH 7.4 PBS at 37 °C. All of the Raman spectra were obtained using a Renishaw inVia-Reflex Raman spectrometer equipped with a 785 nm excitation laser.

### Cell culture

The HeLa cells were cultured in Dulbecco’s Modified Eagles’ Medium (DMEM, KeyGEN BioTECH), supplemented with 10% fetal bovine serum (FBS, Life), and 1% antimycotic solution (KeyGEN BioTECH) at 37 °C in a humidified incubator containing 5% CO_2_ and 95% air.

### Preparation of cell samples for SERS measurement

Cell lysis was obtained by cells treated with NP-40 lysis (KGP705, KeyGEN BioTECH), and then reacted with PRPs. The pH of cell lysis was adjusted by 0.1 M NaOH to 7.4. For single living cells, the cells were pre-cultured in 12-well cell culture plates for 24 h and to these 0.5 nM PRPs were added. After incubation for 0.5 h at 4 °C, the cells were incubated at 37 °C for another 2 h to allow the internalization of PRPs, and then washed with pH 7.4 PBS three times. Then the coverslips were placed in a homemade live cell chamber with pH 7.4 PBS maintained stable humidity and 37 °C temperature.

### Finite-difference time-domain (FDTD) simulation

The electromagnetic field distribution of the AuNS was simulated by FDTD *via* a commercial software package (Lumerical Solutions, Inc.). The dielectric constant of gold was from Johnson and Christy.^41^ The computational domain was bounded by perfectly matched layer (PML) and the AuNS was excited with a quasi nonpolarized light, which consisted of an *x*-polarized and *y*-polarized incident plane wave propagating along the *z*-axis.

### Mathematic model and data analysis

We developed a quantitative principle component analysis (qPCA) method to quantify each of the components in a mixed system. The full proof and details about the mathematical model and calculation strategy are provided in the ESI.† Based on the above qPCA, all the data processing and calculation in this workflow is programmed, facilitating efficient proportion calculation. To do this, the Raman spectra were processed and analyzed using MATLAB R2015b. Prior to PCA, 20 of the spectra from each group (Cys product, Hcy product, GSH product and mixed product) were randomly selected and each spectrum implemented area normalization. The scatter plot of the PC1 *vs.* PC2 scores was utilized to classify the four products.

## Conflicts of interest

There are no conflicts to declare.

## Supplementary Material

Supplementary information
