# Supplementary material for: Simultaneous quantification of multiple endogenous biothiols in single living cells by plasmonic Raman probes
Source: Chem Sci. 2017 Aug 29;8(11):7582–7. doi: 10.1039/c7sc03218h (PMC5848793; doi:10.1039/c7sc03218h)
Supplement: Supplementary file 1 [file SC-008-C7SC03218H-s001.pdf]

Electronic Supplementary Material (ESI) for Chemical Science.
This journal is © The Royal Society of Chemistry 2017

Electronic Supplementary Information

Simultaneously quantify multiple endogenous biothiols in single living cells by plasmonic Raman probes

Shan-Shan Li,[⊥] Qi-Yuan Guan,[⊥] Mengmeng Zheng,[⊥] Yu-Qi Wang, Deju Ye, Bin Kang,* Jing-Juan Xu* and Hong-Yuan Chen*

State Key Laboratory of Analytical Chemistry for Life Science and Collaborative Innovation Center of Chemistry for Life Sciences, School of Chemistry and Chemical Engineering, Nanjing University, 210023, China

Content:

1. Mathematical model and calculation strategy
2. Strategy for discriminating and quantifying Cys, Hcy and GSH
3. Additional schemes, figures and tables

1. Mathematical model and calculation strategy

2.1 Mathematical model

Assume that the mixture is formed by *n* constituents and each has *k* detecting variables, the spectra of both pure constituents and mixture could be stored as a row vector whose columns are intensities at certain data point of “x-axis”. Then we have the following two equations (eq. S1 and eq. S2):

$$M = \sum_{n}^{i=1} K_i C_i \qquad (S1)$$

$$\sum_{n}^{i=1} K_i = 1 \qquad (S2)$$

where *M* is the row vector stands for the mixture spectrum and *C* is a matrix whose rows are spectra of constituents of the mixture with a corresponding proportions array, written as a column vector *K*, the index *i* refers to the *ith* row of the variable. According to above two basic equations, *K* can be evaluated if *C* and *M* are provided.

For ideal spectral data, eq. S1 and eq. S2 are valid and with the relationship between spectra of constituents and that of their mixture, we can easily rewrite these two equations as (eq. S3 and eq. S4):

$$C^T K = M^T \qquad (S3)$$

$$su\, K = 1 \qquad (S4)$$

where *K* is a column vector of proportion of each constituent within the mixture and *su K* is the sum of elements within *K*. The upper *T* means transpose. Then *C* and *M* are arranged vertically and stored as a matrix *A*, and PCA algorithm is applied to *A* to obtain principal component scores.

A regular PCA procedure has two main steps that alter the original data: zero-mean step and projection step. For zero-mean step, the column-means are subtracted from *A* to form a new matrix *B* whose mean values of each column are all zero. Thus, we have (eq. S5):

$$B = A - \bar{A} \tag{S5}$$

where $\bar{A}$ is a row vector whose value of each column is the mean of the same column within *A*. Please note that when only row size or column size of two matrixes is the same, the minus operation in this equation performs a subtraction that subtracts subtrahend with every columns or rows of the minuend respectively in turn, and the result is a matrix which is of the same size with the minuend. From eq. S3, eq. S4 and eq. S5, we have (eq. S6):

$$(C - \bar{A})^T K = (M - \bar{A})^T \tag{S6}$$

Equation above clearly show that the original *K* still fits eq. 3 after *C* and *M* are zero-meant. The projection step calculates PC scores with the new matrix *A* which is zero-meant, we have (eq. S7):

$$S_A = AL = \begin{bmatrix} S_C \\ S_M \end{bmatrix} = \begin{bmatrix} CL \\ ML \end{bmatrix} \tag{S7}$$

where S_A is the matrix of PC scores and *L* is corresponding set of eigenvectors called loadings. *CL* (S_C) and *ML* (S_M) are PC scores of *C* and *M* after projection, respectively. The brackets are used to form matrices. From eq. S3 we have (eq. S8):

$$(CL)^T K = (ML)^T \tag{S8}$$

The eq. S8 could be further expressed as (eq. S9 and eq. S10):

$${S_C}^T K = {S_M}^T \tag{S9}$$

$$K = {S_C}^T \backslash {S_M}^T \qquad (S10)$$

which demonstrated that K can be evaluated with PC scores just as with the original spectra data.

2.2 Calculation of proportion array *K*.

In PC space, the distance of two sample points within a PC scores plot reflects how similar the two samples are and vice versa. A shorter distance between a mixture and its constituent means a higher proportion of this constituent. For a system of two constituents and their mixture, after spectra acquisition, we can map the spectra into an axis with their PC1 scores depicted below and calculate the composition of the mixture with these two equations: (eq. S11 and eq. S12):

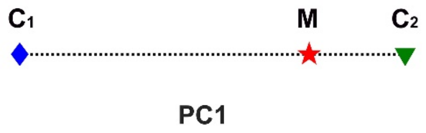

$$k_{C_1} = \frac{MC_2}{C_1C_2} \qquad (S11)$$

$$k_{C_2} = \frac{MC_1}{C_1C_2} \qquad (S12)$$

where k_{C1} and k_{C2} are the proportions of constituent C_1 and C_2 to form mixture M, respectively.

Likewise, for a system of three constituents and their mixture, we can map the spectra with their PC1 and PC2 scores into a plane:

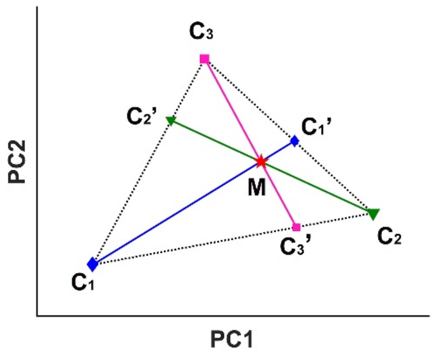

We can consider the mixture M to be formed in two steps: (1) mixing C_1 and C_2 into C_3', (2) mixing C_3' and C_3 into M. Thus proportions can be resolved by doing math with eq. S11 and eq. S12 twice. The final equations to calculate proportions of each of these three constituents are (eq. S13, eq. S14 and eq. S15):

$$k_{C_1} = \frac{MC_1'}{C_1C_1'} \qquad (S13)$$

$$k_{C_2} = \frac{MC_2'}{C_2C_2'} \qquad (S14)$$

$$k_{C_3} = \frac{MC_3'}{C_3C_3'} \qquad (S15)$$

where C_1', C_2' and C_3' are intersection points of line C_2C_3 and MC_1 extension line, line C_1C_3 and MC_2 extension line, line C_1C_2 and MC_3 extension line, respectively. Validity of these three equations can be illustrated below.

Assume that A, B and C are constituents with proportions of P_A, P_B and P_C in mixture O, respectively, and their relationship can be illustrated below.

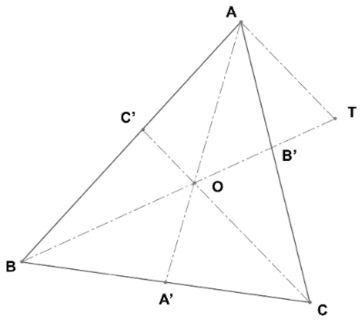

With knowledge of triangular coordinate and two-component system described in the

manuscript, we can easily have

$$\frac{P_A}{P_B} = \frac{C'B}{C'A} \tag{S16}$$

$$\frac{P_A}{P_C} = \frac{B'C}{B'A} \tag{S17}$$

With auxiliary line AT//CC', the proportion of C within O can be calculated with

$$P_C = \frac{P_C}{P_A + P_B + P_C} = \frac{1}{1 + \frac{P_B}{P_C} + \frac{P_A}{P_C}} = \frac{1}{1 + \frac{P_A}{P_C} \times \left(1 + \frac{P_B}{P_A}\right)}$$

$$= \frac{1}{1 + \frac{B'C}{B'A} \times \frac{AB}{C'B}} = \frac{1}{1 + \frac{OC}{AT} \times \frac{AT}{C'O}} = \frac{1}{1 + \frac{OC}{C'O}} = \frac{C'O}{C'O + OC} = \frac{C'O}{C'C} \tag{S18}$$

Similarly,

$$P_B = \frac{B'O}{B'B} \tag{S19}$$

$$P_A = \frac{A'O}{A'A} \tag{S20}$$

To calculate a mixture containing *n* constituents, its PC1 to PC*n*-1 scores were needed to be mapped in an *n-1* dimensional space and the proportion of each constituent could be calculated according to *n* equations similar to eq. S16 - eq. S20. For a system with *n* (*n*≥2) constituents, after spectra acquisition and PCA procedure, we can scratch scores of first *n-1* PCs and calculate *K* with

$$K_{n\times1} = [S_C\ \boldsymbol{e}]^T \backslash [S_M\ 1]^T \tag{S21}$$

where $\boldsymbol{e}$ is an all-ones column vector with a same number of rows as S_C and $K_{n\times1}$ indicates a column vector with *n* rows. Every piece of data processing and calculation in this workflow is programmable, facilitating efficient proportion calculation without any spectra inspection or peak attribution.

2. **Strategy for discriminating and quantifying Cys, Hcy and GSH**

For a reaction: $A + B \rightarrow C$, its reaction rate (r) could be given by:

$$r = -\frac{d[A]}{dt} = k[A][B] \tag{S22}$$

where k is the second-order reaction rate constant. If one reactant is of large excess over the other (e.g. [B]>>[A]), then eq. 22 could be written as:

$$r = k[A][B] = k'[A] \tag{S23}$$

where $k' = k[B]_0$, k' is the pseudo-first-order reaction rate constant, and $[B]_0$ is the initial concentration of B. By collecting k' for different concentrations of [B], a plot of k' vs [B] gives k as the slope.

In our current work, PRPs react with Cys, Hcy or GSH at the same time. If only tiny PRPs were used, that is, [Cys], [Hcy] and [GSH] >> [PRPs], we can obtain:

$$\begin{aligned} r_{PRPS} &= r_{Cys} + r_{Hcy} + r_{GSH} \\ &= [PRPs]\left(k'_{Cys} + k'_{Hcy} + k'_{GSH}\right) \\ &= [PRPs]k'_{mix} \end{aligned} \tag{S24}$$

$$\begin{aligned} k'_{mix} &= k'_{Cys} + k'_{Hcy} + k'_{GSH} \\ &= k_{Cys}[Cys]_0 + k_{Hcy}[Hcy]_0 + k_{GSH}[GSH]_0 \end{aligned} \tag{S25}$$

If $[Cys]_0 : [Hcy]_0 : [GSH]_0 = x : y : z$, the above formula can be converted into:

$$k'_{mix} = \left(k_{Cys} + k_{Hcy}\frac{y}{x} + k_{GSH}\frac{z}{x}\right)[Cys]_0 \tag{S26}$$

According to eq. S26, for a mixed system with unknown $[Cys]_0$, $[Hcy]_0$ and $[GSH]_0$, with second-order rate constants k_{Cys}, k_{Hys} and k_{GSH}, which do not depend on the concentration of reactants, the pseudo-first-order hybrid reaction rate constant k'_{mix} could be experimentally determined by monitoring the reaction kinetics of PRPs with

the mixture of Cys, Hcy and GSH. If $x:y:z$ was got, we could calculate $[Cys]_0$, $[Hcy]_0$ and $[GSH]_0$, ultimately. To obtain $x:y:z$, we developed qPCA method to calculate $x:y:z$ from the Raman spectral features of three products of PRPs reacted with Cys, Hcy or GSH.

3. Additional figures and tables

Scheme S1 Synthesis of Raman reporter **TA-CBT**. Reaction conditions (a): MMP, isobutyl chloroformate, THF, 0 °C to room temperature, 74%.

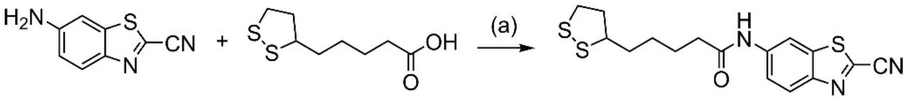

TA-CBT

Scheme S2 Schematic diagram explaining the workflow.

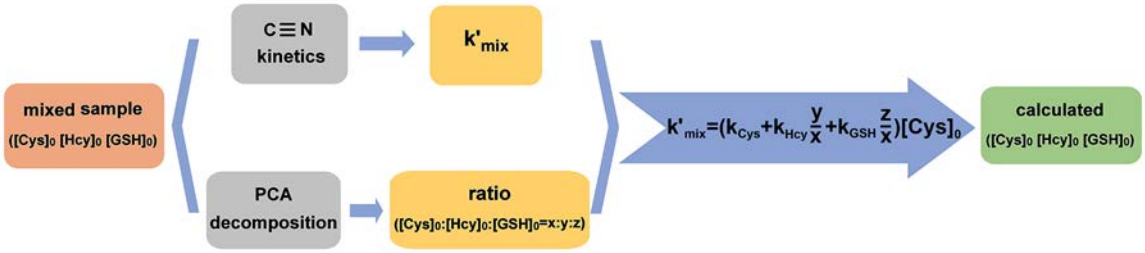

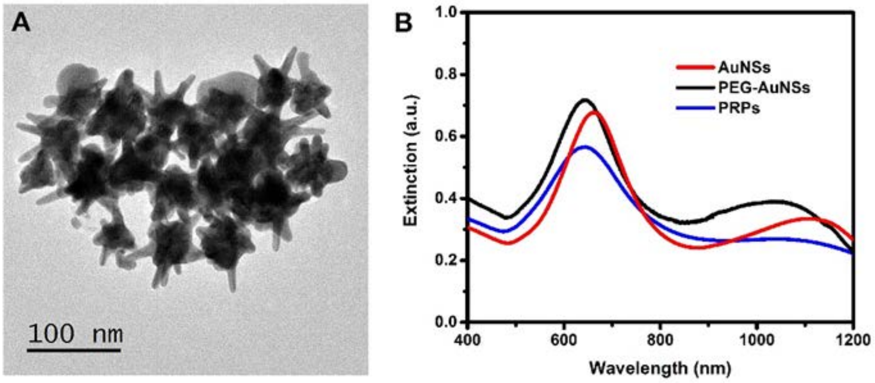

Fig. S1 (A) TEM micrograph of AuNSs. (B) UV-vis extinction spectra of AuNSs before (red) and after conjugation with PEG (black), and PRPs (blue).

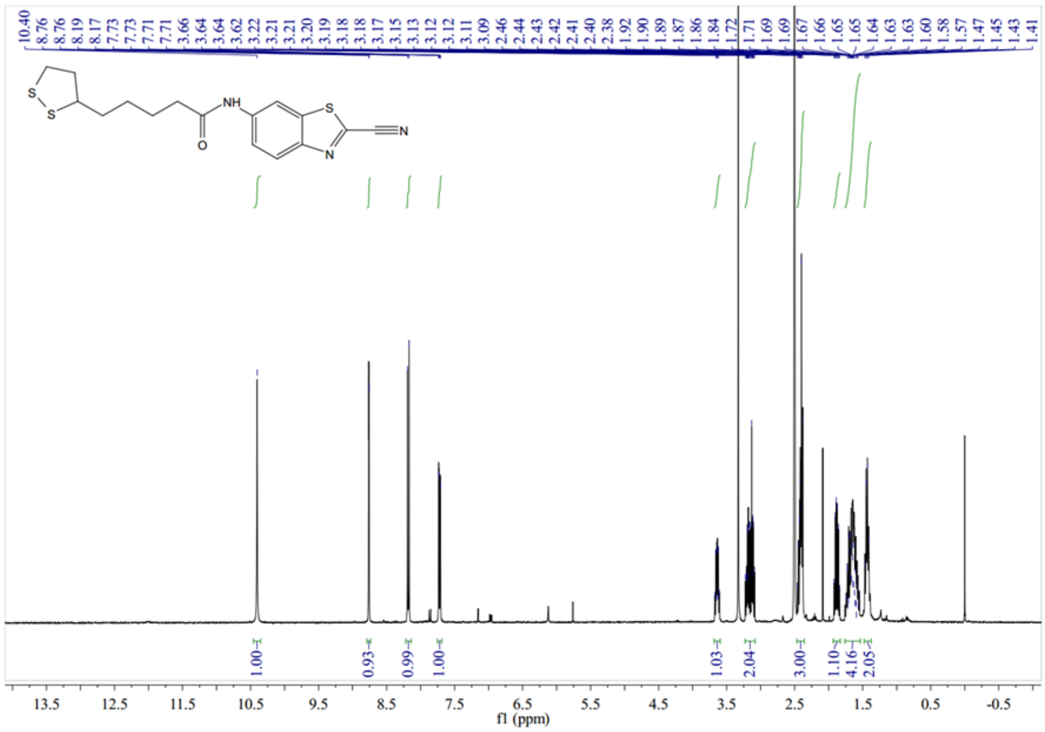

Fig. S2 ^{1}H NMR spectra of Raman reporter **TA-CBT**.

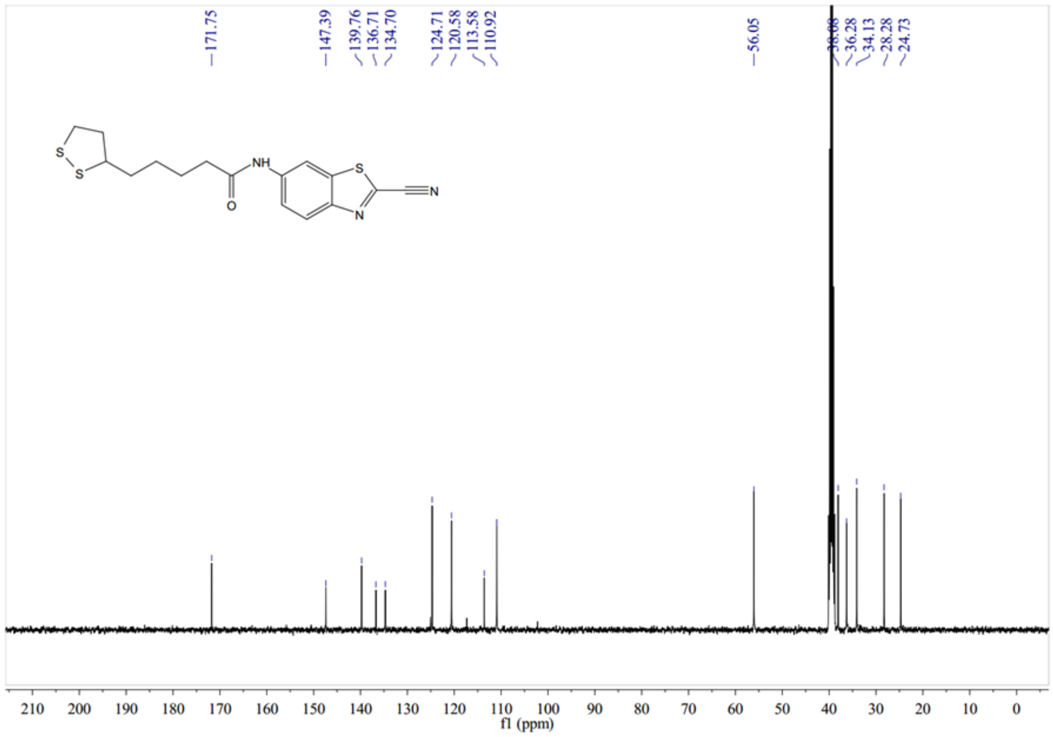

Fig. S3 ^{13}C NMR spectra of Raman reporter **TA-CBT**.

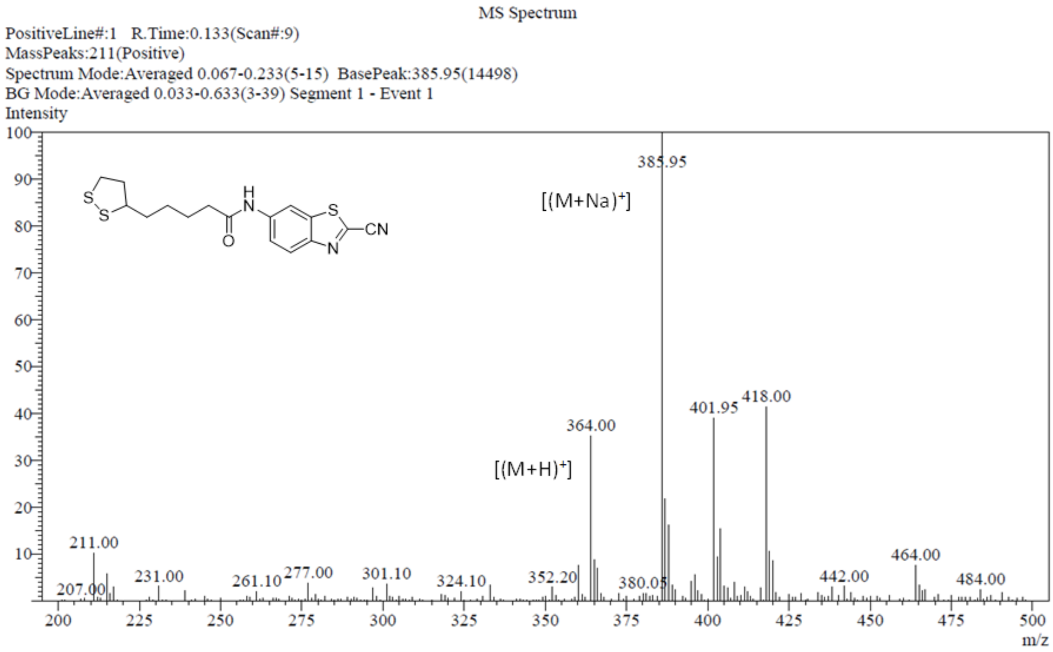

Fig. S4 LC-MS spectra of Raman reporter **TA-CBT**.

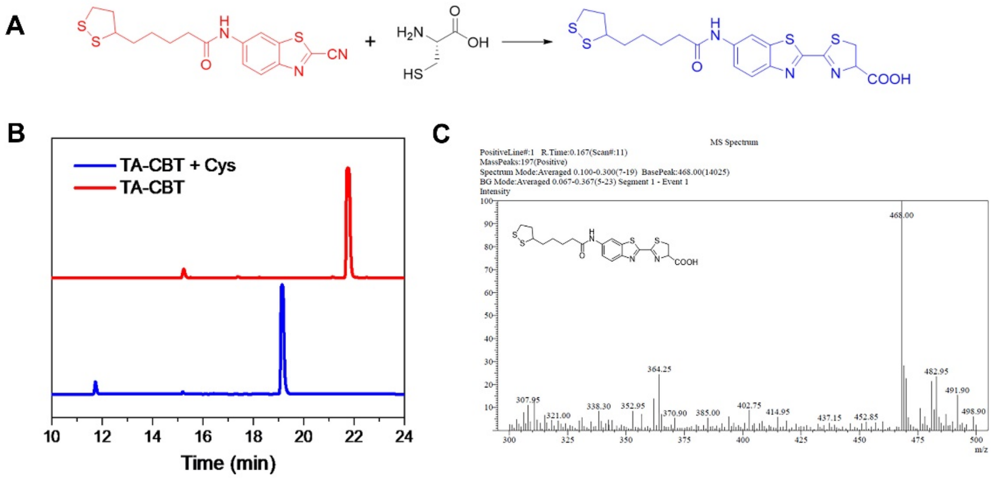

Fig. S5 (A) The reaction of **TA-CBT** with Cys. (B) HPLC spectra of Raman reporter **TA-CBT** and the reaction solution of **TA-CBT** (1 mM) with Cys (10 mM) at 37 °C for 1 h. (C) LC-MS analysis of the product. The product is calcd. for $C_{19}H_{22}N_3O_3S_4^+$ $[(M+H)^+]$: 468.05; found 468.00.

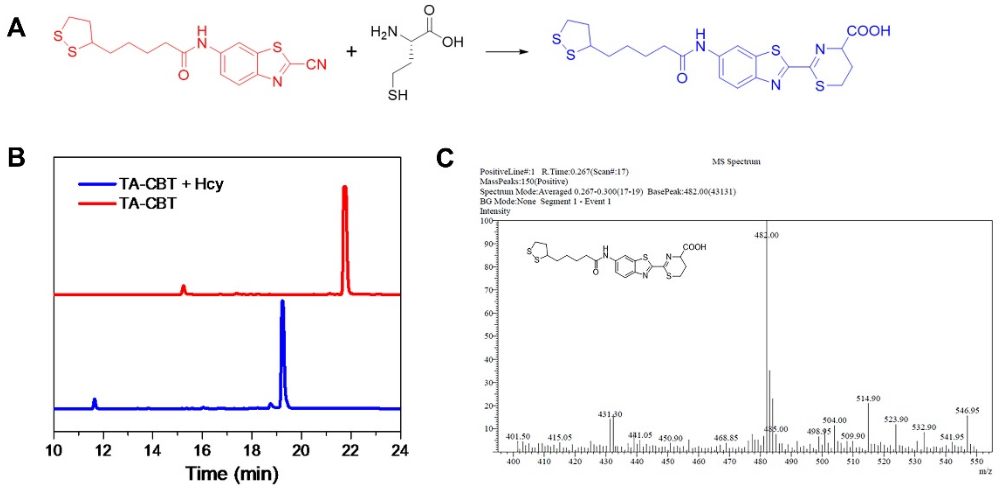

Fig. S6 (A) The reaction of **TA-CBT** with Hcy. (B) HPLC spectra of Raman reporter **TA-CBT** and the reaction solution of **TA-CBT** (1 mM) with Hcy (10 mM) at 37 °C for 1 h. (C) LC-MS analysis of the product. The product is calcd. for $C_{20}H_{24}N_3O_3S_4^+$ $[(M+H)^+]$: 482.07; found 482.00.

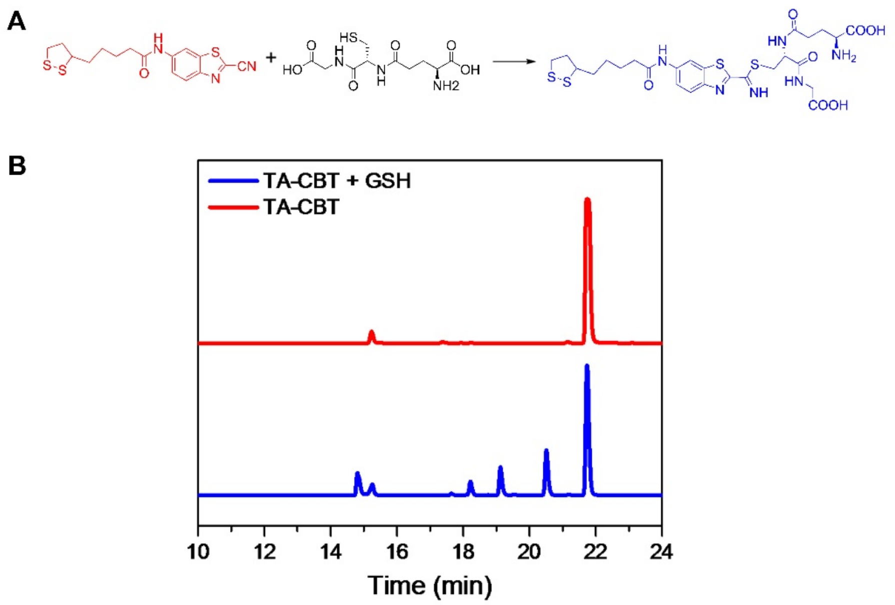

Fig. S7 (A) The reaction of **TA-CBT** with GSH. (B) HPLC spectra of Raman reporter **TA-CBT** and the reaction solution of **TA-CBT** (1 mM) with GSH (10 mM) at 37 °C for 1 h.

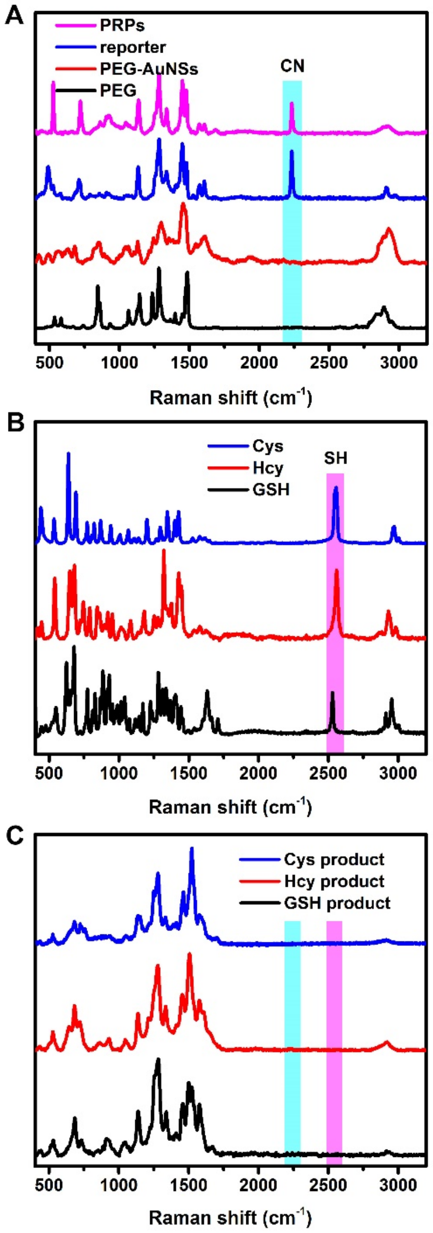

Fig. S8 Raman spectra of PRPs (A), three biothiols (B), and three products (C).

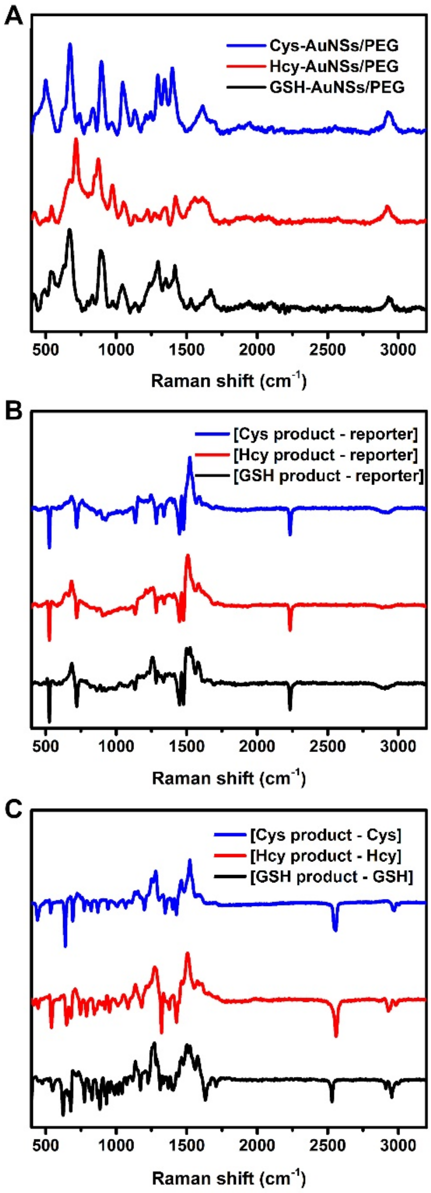

Fig. S9 (A) Raman spectra of Cys, Hcy and GSH modified AuNSs/PEG. (B and C) The difference spectra, e.g. [Cys product - Cys] was defined as a product minus a reactant.

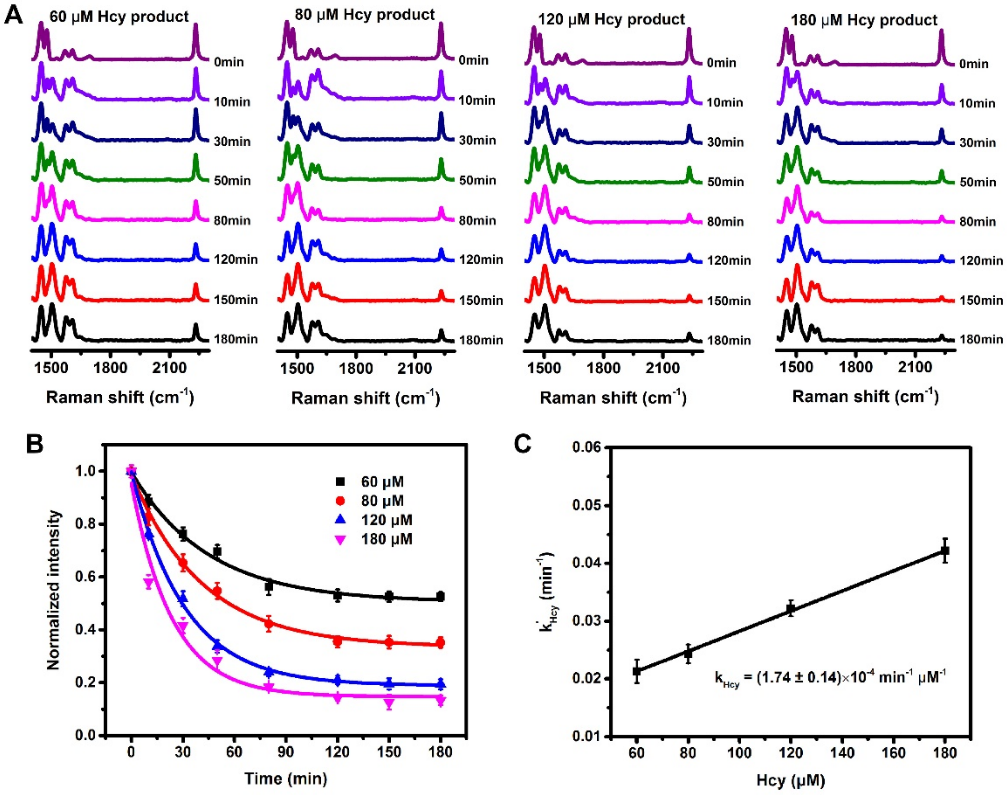

Fig. S10 (A) Raman spectra of PRPs reacted with different concentrations (60, 80, 120 and 180 μM) of Hcy in pH 7.4 PBS at 37 °C for 180 min. (B) Trends of C≡N over time at different concentrations of Hcy. (C) Pseudo first-order rate constant against concentration of Hcy.

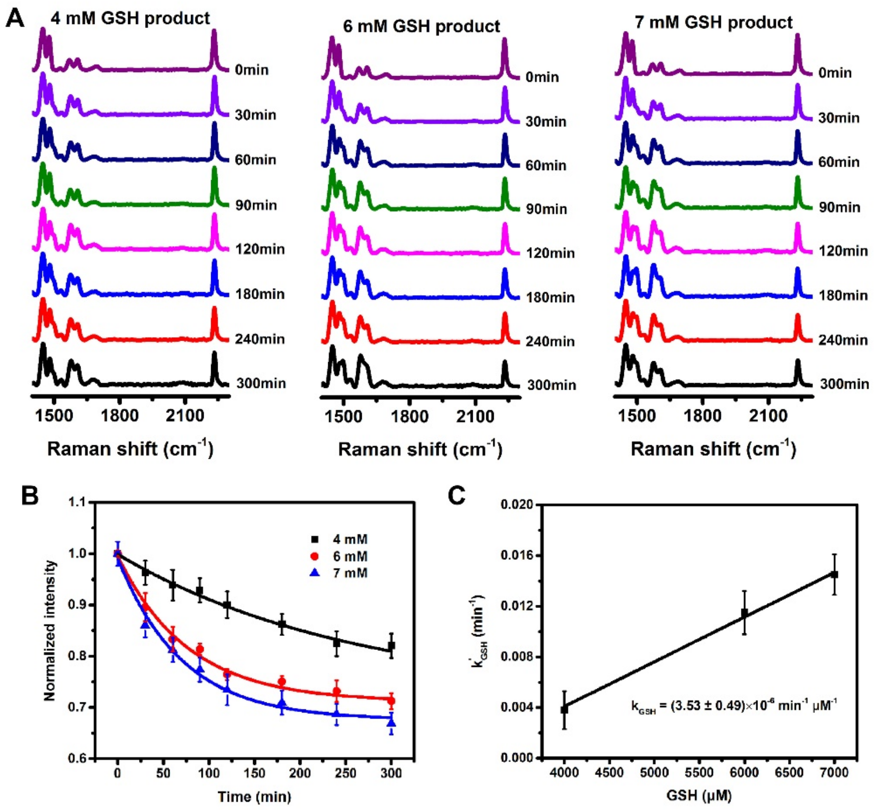

Fig. S11 (A) Raman spectra of PRPs reacted with different concentrations (4, 6 and 8 mM) of GSH in pH 7.4 PBS at 37 °C for 300 min. (B) Trends of C≡N over time at different concentrations of GSH. (C) Pseudo first-order rate constant against concentration of GSH.

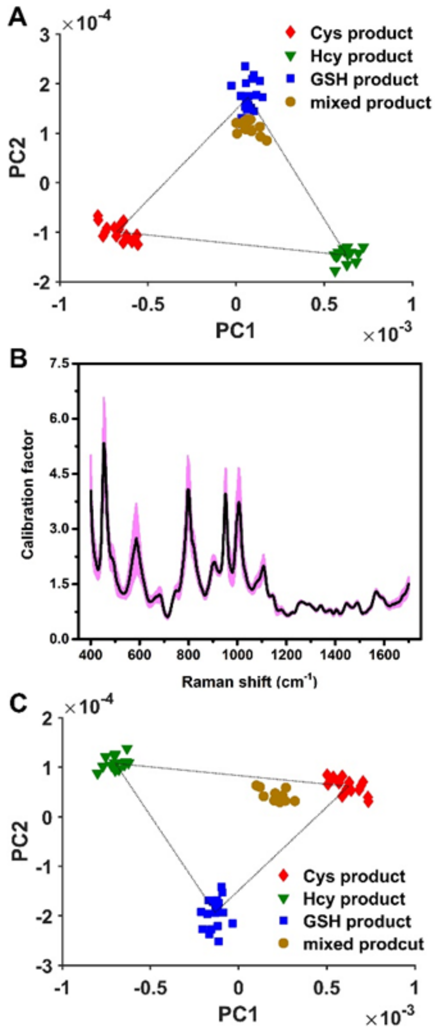

Fig. S12 Scatter plot of PC1 vs PC2 scores of Cys product, Hcy product, GSH product and mixed product before (A) and after (C) calibration. (B) Calibration curve for three biothiols: the averaged calibration curve from 20 curves were shown as black line and the standard deviation was highlighted as pink color.

In our current work, PRPs can react with Cys, Hcy and GSH at the same time obtaining three different products with a wealth of fingerprint information in Raman spectra, but it is difficult to differentiate the one from the other by visual. In order to extract the most principal differences, we analyzed the data using PCA on the Raman spectra of

the three products. Taking into consideration of the intracellular concentration of Cys, Hcy and GSH, we gave the initial concentrations of the three biothiols and then the given ratio of the three biothiols could be got in simulation experiment (Table S2). Because the given initial concentration ratio of the three biothiols ($[Cys]_0 : [Hcy]_0 : [GSH]_0 = x : y : z$) was different from the weight of the three products in a mixed product (Table S2 and Fig. S12A), so we used spectral mixture of the three products to rectify the discrepancy. Based on the given ratio of the three biothiols and the Raman spectra of the three products, we could get a spectral mixture. The calibration curve for three biothiols was derived from a spectral mixture divided by a mixed product (Fig. S12B), so if giving a mixed product, we could calculate a corresponding spectral mixture, utilizing qPCA, thus the initial concentration ratio of the three biothiols could be calculated (Fig. S12C).

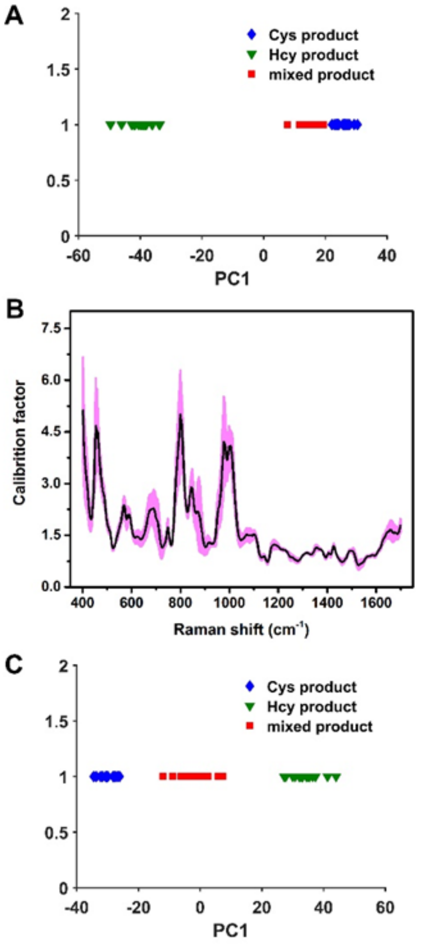

Fig. S13 Scatter plot of PC1 scores of Cys product, Hcy product and mixed product before (A) and after (C) calibration. (B) Calibration curve for two biothiols: the averaged calibration curve from 20 curves were shown as black line and the standard deviation was highlighted as pink color.

For two component system, to acquire the initial concentration ratio of the two biothiols ($[Cys]_0 : [Hcy]_0 = x : y$), we used spectral mixture of two products to rectify the discrepancy (Table S3 and Fig. S13). Based on the given ratio of the two biothiols and the Raman spectra of the two products, we could get a spectral mixture. The calibration curve for two biothiols was derived from a spectral mixture divided by a mixed product

(Fig. S13B), so if giving a mixed product, we could calculate a corresponding spectral mixture, utilizing qPCA, thus the initial concentration ratio of the two biothiols could be calculated (Fig. S13C).

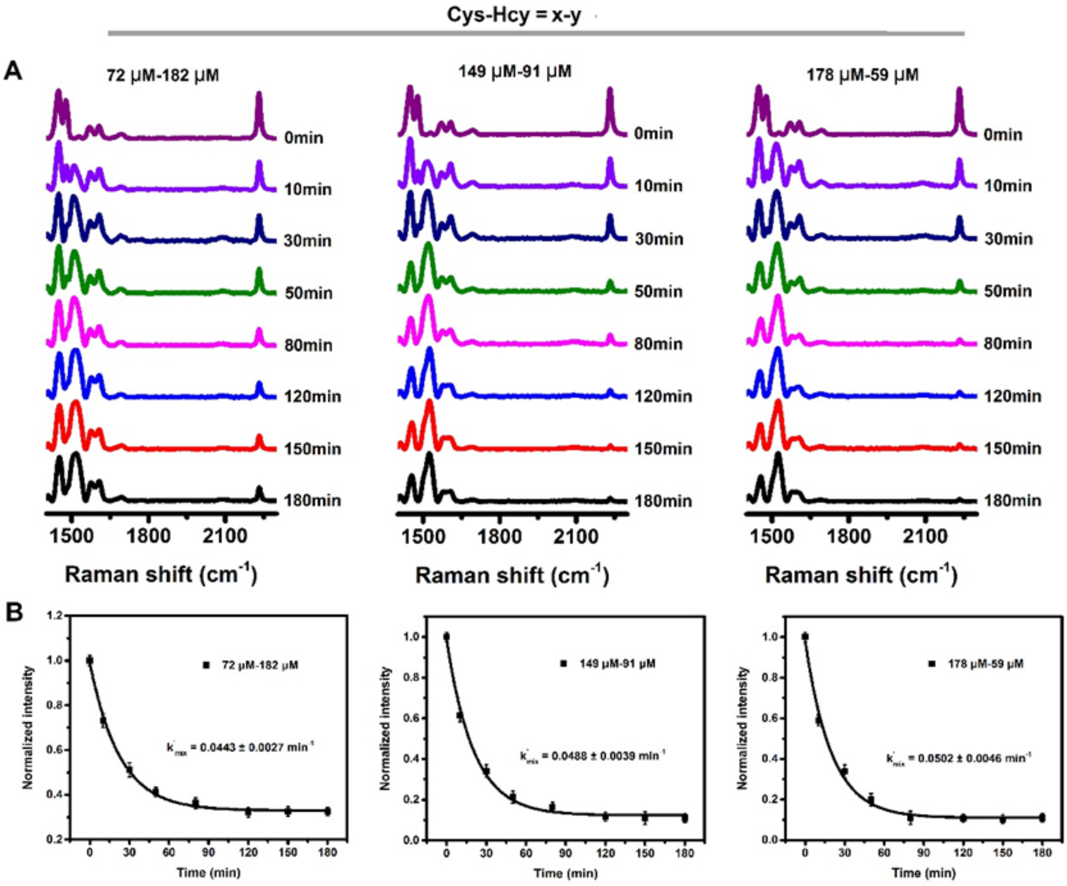

Fig. S14 (A) Raman spectra of PRPs reacted with different concentrations of Cys and Hcy in pH 7.4 PBS at 37 °C for 180 min. (B) Trends of C≡N over time at different concentrations of Cys and Hcy.

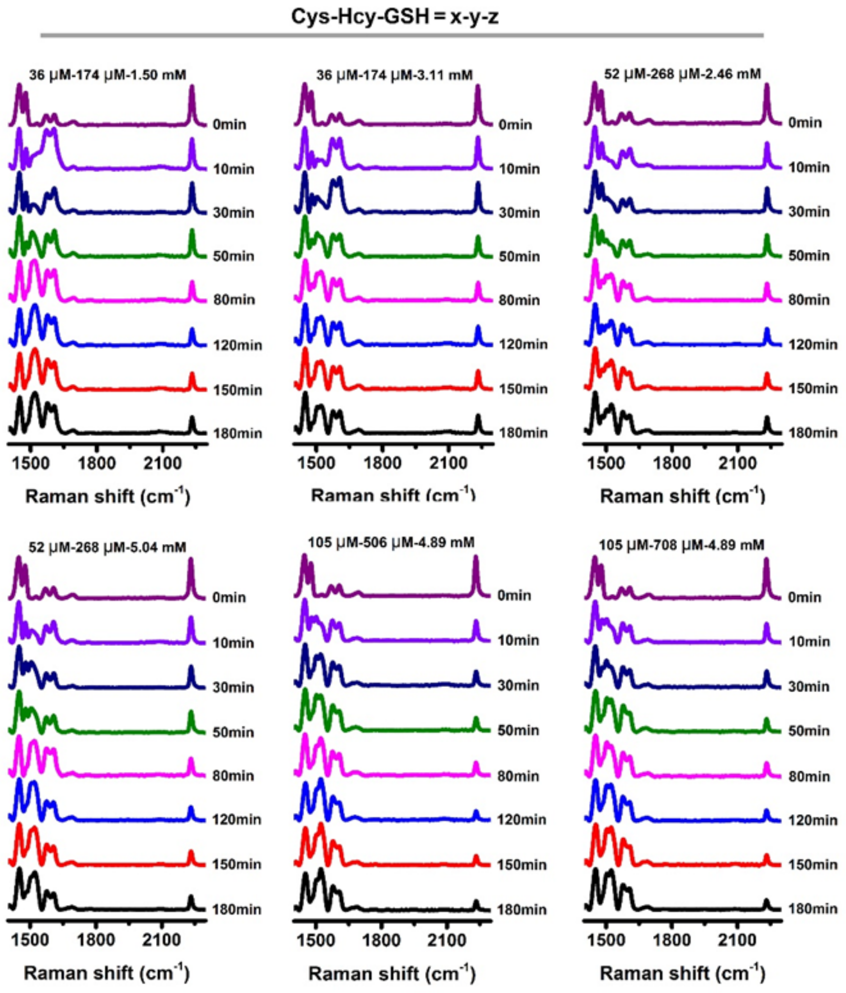

Fig. S15 Raman spectra of PRPs reacted with different concentrations of Cys, Hcy and GSH in pH 7.4 PBS at 37 °C for 180 min.

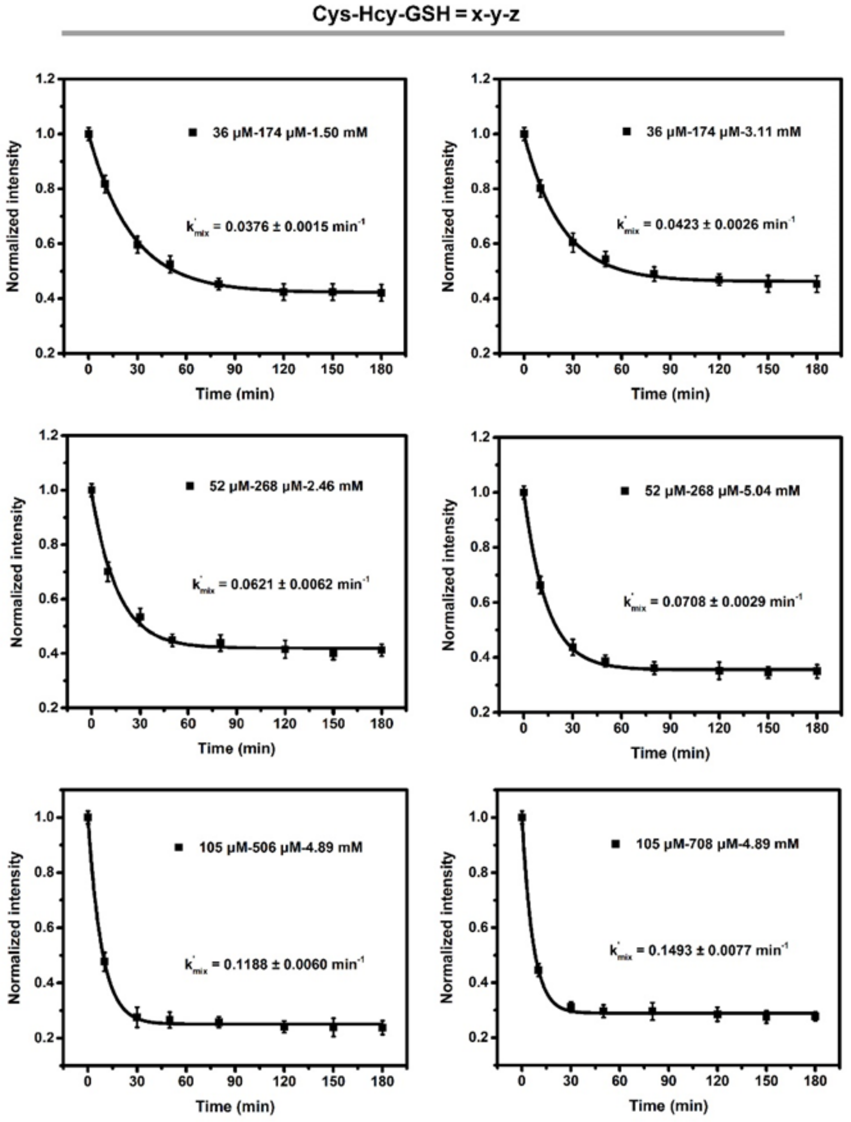

Fig. S16 Trends of C≡N over time at different concentrations of Cys, Hcy and GSH.

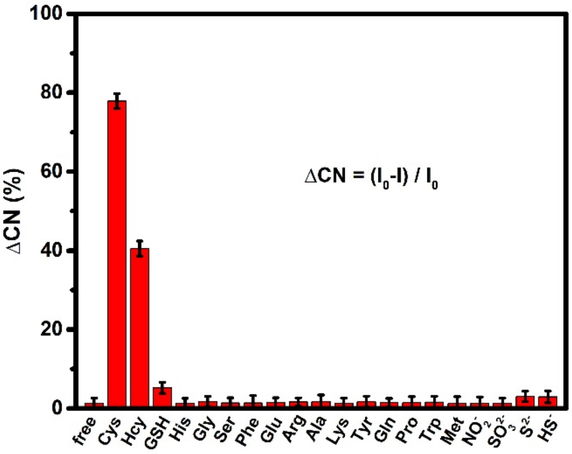

Fig. S17 The changes of △C≡N in the presence of 100 μM diverse amino acids (free, Cys, Hcy, GSH, His, Gly, Ser, Phe, Glu, Arg, Ala, Lys, Tyr, Gln, Pro, Trp and Met) and possible necleophiles (NO_2^-, SO_3^{2-}, S^{2-} and HS^-) in pH 7.4 PBS at 37 °C for 180 min.

To examine the reaction selectivity, PRPs were treated with biologically related amino acids and possible necleophiles (Fig. S17). No observable changes of C≡N were observed with other amino acids and necleophiles (NO_2^-, SO_3^{2-}), but a little change with S^{2-} and HS^-. Considering the reactivity and the intracellular concentrations of Cys (30~200 μM), Hcy (0.1~1 mM) and GSH (1~10 mM) reported in previous literatures,[1-6] the average intracellular H_2S level is in sub-micromolar range,[7-9] which is produced in cells through enzymatic, non-enzymatic processes and exogenous added.[7-10] Thus, in our current system, the sub-micromolar intracellular H_2S seems not to have obvious influence on quantifying these three biothiols with either much more higher concentration (GSH) or much more higher reactivity (Cys and Hcy) than H_2S.

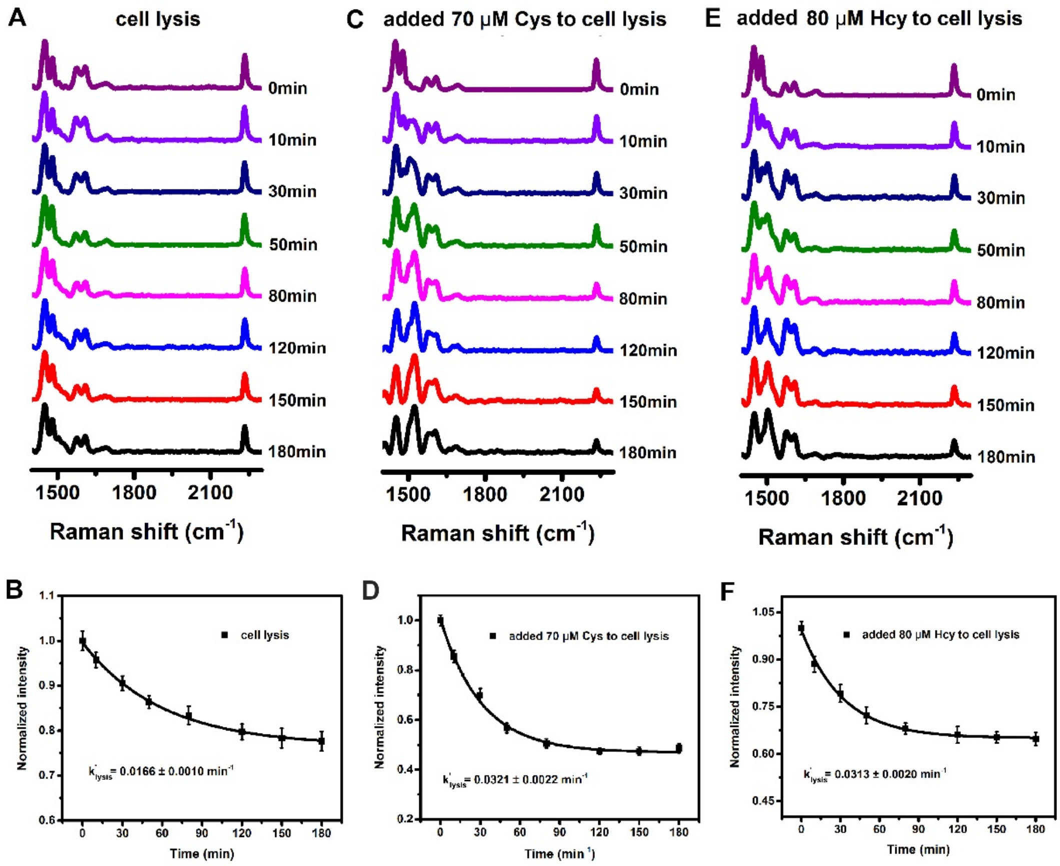

Fig. S18 Raman spectra of PRPs reacted with Cys, Hcy and GSH in cell lysis (A), with the addition of 70 μM Cys (C), and 80 μM Hcy (E) to cell lysis for 180 min. Trends of C≡N over time for cell lysis (B), with the addition of 70 μM Cys (D), and 80 μM Hcy (F) to cell lysis.

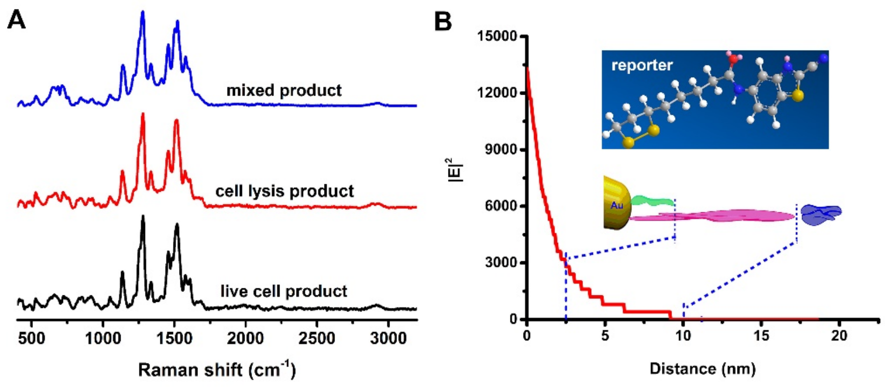

Fig. S19 (A) Raman spectra of mixed product (blue), cell lysis product (red), and live cell product (black). (B) Localized electronic field of AuNS against distance.

From Fig. S19A, we could see that the Raman spectra of three product samples were quite similar with each other demonstrating that our experimental results were reliable. To illustrate this point, we extracted localized electronic field of AuNS from FDTD simulation result shown in Fig. S19B and $|E|^2$ reduce rapidly. On the basis of bond lengths, we estimated the lengths of reporter (~2.5 nm) and PEG (~10 nm), which were in accordance with the hydrodynamic diameters (Table S1). Based on the above, we can say that the signal enhancement of Raman spectra is mainly derived from reporter, so the spectra of three product samples were quite similar with each other.

Table S1 Hydrodynamic diameter of AuNSs, PEG-AuNSs and PRPs.

Sample	Hydrodynamic diameter (nm)	Polydispersity
AuNSs	52.78 ± 0.58	0.32
PEG-AuNSs	73.75 ± 0.38	0.31
PRPs	71.61 ± 1.76	0.27

Table S2 The ratio of Cys, Hcy and GSH before and after calibration.

No.	Given concentration			Given ratio			Calculated ratio		
	Cys (μM)	Hcy (μM)	GSH (mM)	Cys (%)	Hcy (%)	GSH (%)	Cys (%)	Hcy (%)	GSH (%)
1	64	333	3.02	1.88	9.74	88.38	2.10	10.83	87.07
2	64	466	3.02	1.81	13.12	85.06	1.51	12.13	86.36
3	64	639	3.02	1.73	17.16	81.11	2.03	15.40	82.57
4	129	333	3.02	3.70	9.56	86.74	5.25	8.58	86.17
5	129	466	3.02	3.56	12.89	83.55	2.71	15.58	81.71
6	129	639	3.02	3.40	16.87	79.73	4.47	15.96	79.57
7	64	333	6.00	1.01	5.21	93.79	1.15	5.81	93.04
8	64	466	6.00	0.99	7.14	91.87	1.53	8.06	90.41
9	64	639	6.00	0.96	9.54	89.50	1.34	10.50	88.16
10	129	333	6.00	1.99	5.16	92.85	2.46	6.67	90.87
11	129	466	6.00	1.95	7.07	90.97	1.94	6.25	91.81
12	129	639	6.00	1.90	9.45	88.65	3.09	10.52	86.39
Mean absolute error (%)							0.59	1.19	1.27

Table S3 The ratio of Cys and Hcy before and after calibration.

No.	Given concentration		Given ratio		Calculated ratio	
	Cys (μM)	Hcy (μM)	Cys (%)	Hcy (%)	Cys (%)	Hcy (%)
1	124	133	48.17	51.83	50.48	49.52
2	124	266	31.73	68.27	35.08	64.92
3	123	400	23.54	76.46	28.21	71.79
4	372	133	73.61	26.39	78.89	21.11
5	372	666	35.81	64.19	30.35	69.65
6	619	400	60.77	39.23	65.36	34.64
Mean absolute error (%)					4.27	4.27

Table S4 The simulation experimental calculated results in vitro.

No.	Given concentration			Given ratio			Calculated ratio			Calculated k'$_{mix}$ (min^{-1}) 37 °C	Calculated concentration		
	Cys (μM)	Hcy (μM)	GSH (mM)	Cys (%)	Hcy (%)	GSH (%)	Cys (%)	Hcy (%)	GSH (%)		Cys (μM)	Hcy (μM)	GSH (mM)
1	36	174	1.50	2.11	10.18	87.71	2.25	11.32	86.43	0.0376	31	153	1.19
2	36	174	3.11	1.08	5.25	93.67	1.23	5.84	92.93	0.0423	32	152	2.42
3	52	268	2.46	1.87	9.64	88.49	2.09	10.72	87.19	0.0621	49	251	2.05
4	52	268	5.04	0.97	5.00	94.03	1.11	5.58	93.31	0.0708	51	257	4.29
5	105	506	4.89	1.91	9.20	88.89	2.13	10.23	87.64	0.1188	99	475	4.07
6	105	708	4.89	1.84	12.41	85.74	1.53	11.47	87.00	0.1493	86	648	4.89
Average relative error (%)											9.42	8.29	15.19

Table S5 The calculated results for two biothiols system.

No.	Given concentration (μM)		Given ratio (%)		Calculated ratio (%)		Calculated k'_{mix} (min^{-1}) 37 °C	Calculated concentration (μM)	
	Cys	Hcy	Cys	Hcy	Cys	Hcy		Cys	Hcy
1	72	182	28.35	71.65	28.21	71.79	0.0443	67	170
2	178	59	75.11	24.89	78.89	21.11	0.0502	187	50
3	149	91	62.08	37.92	65.36	34.64	0.0488	155	82
Average relative error (%)								5.34	10.58

References

1 J. Yin, Y. Kwon, D. Kim, D. Lee, G. Kim, Y. Hu, J.-H. Ryu and J. Yoon, *J. Am. Chem. Soc.*, 2014, **136**, 5351.

2 A. Meister, *J Biol Chem*, 1988, **263**, 17205.

3 K. Ock, W. I. Jeon, E. O. Ganbold, M. Kim, J. Park, J. H. Seo, K. Cho, S.-W. Joo and S. Y. Lee, *Anal. Chem.*, 2012, **84**, 2172.

4 S. Park and J. A. Imlay, *J. Bacteriol.*, 2003, **185**, 1942.

5 J. Liu, Y.-Q. Sun, Y. Huo, H. Zhang, L. Wang, P. Zhang, D. Song, Y. Shi and W. Guo, *J. Am. Chem. Soc.*, 2014, **136**, 574.

6 M. A. Medina, J. L. Urdiales and M. I. Amores‐Sánchez, *Eur. J. Biochem.*, 2001, **268**, 3871.

7 D.-W. Li, L.-L. Qu, K. Hu, Y.-T. Long and H. Tian, *Angew. Chem. Int. Ed.*, 2015, **54**, 12758.

8 B. Xiong, R. Zhou, J. Hao, Y. Jia, Y. He and E. S. Yeung, *Nat. Commun.*, 2013, **4**, 1708.

9 W. Chen, A. Pacheco, Y. Takano, J. J. Day, K. Hanaoka and M. Xian, *Angew. Chem. Int. Ed.*, 2016, **55**, 9993.

10 V. S. Lin, W. Chen, M. Xian and C. J. Chang, *Chem. Soc. Rev.*, 2015, **44**, 4596.
